# EEG background frequency is associated with discharge outcomes in non-ICU hospitalized patients with COVID-19

**DOI:** 10.3389/fneur.2022.941903

**Published:** 2022-08-29

**Authors:** Kaitlin M. Seibert, Wonhee Lee, Alexandra Eid, Amy E. Espinal, Sara A. Klein, Sumayyah K. Abumurad, James X. Tao, Naoum P. Issa

**Affiliations:** Department of Neurology, University of Chicago, Chicago, IL, United States

**Keywords:** background frequency, COVID-19, discharge outcome, encephalopathy, SARS-CoV-2

## Abstract

**Objective:**

To assess risk factors for encephalopathy in non-ICU hospitalized patients with COVID-19 and the effect of encephalopathy on short-term outcomes.

**Methods:**

We collected clinical and electrophysiological characteristics of fifty patients with COVID-19 infection admitted to a ward service and who had an electroencephalogram (EEG) performed. Associations with short-term outcomes including hospital length of stay and discharge disposition were determined from univariate and multivariate statistical analysis.

**Results:**

Clinical delirium was associated with encephalopathy on EEG, cefepime use was associated with increased length of stay, and of all factors analyzed, background frequency on EEG alone was correlated with discharge disposition.

**Conclusion:**

Encephalopathy is one of the major determinants of short-term outcomes in hospitalized non-ICU patients with COVID-19.

## Key points

- EEG background frequency is independently associated with discharge disposition in hospitalized, non-ICU patients with COVID-19.
- Cefepime use was the only association of hospital length of stay for patients with COVID-19.
- Cefepime use and clinical delirium were independently associated with low EEG background frequency.

## Introduction

Severe acute respiratory syndrome coronavirus 2 (SARS-CoV2) has been associated with several central nervous system (CNS) manifestations including but not limited to anosmia and ageusia, headache, cerebral infarction, cerebral hemorrhage, encephalopathy, and encephalitis (1). The presentation of neurological symptoms has been correlated with severity of the underlying infection. The understanding of SARS-CoV2's overall effect on the neurological system continues to evolve and the exact pathological mechanisms are unclear. The virus triggers an inflammatory response including cytokine storm leading to acute respiratory distress and multi-organ failure, which contributes to hypoxia and metabolic abnormalities. Hypoxia and metabolic abnormalities have previously been shown to frequently present with neurological manifestations (2). The mechanism of neural damage is postulated to be both immune mediated and through cerebrovascular injury (3).

The electroencephalogram (EEG) is useful in the evaluation of patients with impaired consciousness, allowing characterization of the extent, and sometimes the cause, of encephalopathy, assessment for possible non-convulsive seizures and status epilepticus, and assisting with prognostication in certain scenarios (4–6). Encephalopathy is a broad term that implies diffuse dysfunction of brain activity. Electrographically, certain patterns can be seen with encephalopathy including diffuse slowing and periodic discharges (4). In terms of EEG waveform frequency, theta frequencies (4–8 Hz) are seen in mild to moderate degrees of encephalopathy and delta frequencies (<4 Hz) are seen in severe encephalopathy (5). Use of EEG has been a simple, cost-effective approach for assessing infection-associated encephalopathy (6).

There have been case reports documenting presentation of toxic-metabolic encephalopathy, hypoxic encephalopathy, acute hemorrhagic necrotizing encephalopathy and encephalitis associated with COVID-19 infection (3, 7–12). A study in Wuhan China reported that 37% of patients hospitalized with COVID-19 had impaired consciousness (1). Petreseu et al., reported that half the EEG studies in patients infected with SARS-CoV2 were normal, and did not find a difference in fraction of abnormal EEGs between ICU patients and medicine unit patients (13). A retrospective chart review of 42 SARS-CoV2-infected patients found 9 (21.4%) had EEGs with an encephalopathy pattern, defined as continuous or rhythmic frontal or diffuse slow diphasic or triphasic waves or sharp waves (14). In a study by Saez-Landete et al., common EEG characteristics of nonspecific diffuse slowing and low voltage EEG were identified on acute and follow-up EEGs on 15 SARS-CoV2 infected patients (15). Although there have been several studies characterizing encephalopathy and related conditions, there are still gaps in the literature predominantly due to the heterogeneity of small studies whose methods vary widely.

Several studies have examined the prognostic role of EEG findings in ICU patients with COVID-19 (16). Niguet et al., for example, reported a correlation between reactivity on EEG and clinical prognosis, with patients significantly more likely to have a poor outcome if they had an unreactive EEG off sedation (17). However, most patients with COVID-19 do not require ICU care, and there are several factors, such as ICU-induced delirium, commonly found in critical ill patients that can contribute to encephalopathy independent of COVID-19. In this study, we assessed demographic and electrographic characteristics in non-sedated, non-intensive care unit COVID-19 patients to determine if the pattern of activity on EEG is independently associated with clinical outcomes.

## Methods

Medical records were retrospectively reviewed for patients hospitalized between March 1, 2020 to February 28, 2021 at a single institution, University of Chicago Hospital, who tested positive on a SARS-CoV-2 nasal swab (PCR test) and underwent EEG testing. EEG studies were performed using standard international 10–20 system plus supplementary subtemporal electrodes (F9, T9, M1, F10, T10 and M2); in three cases, early in the pandemic, a limited montage consisting of eight channels was used. To minimize the effect of sedating medications on assessment of encephalopathy, patients who were intubated or in an intensive care unit at the time of the EEG were excluded from analysis.

Clinical characteristics and laboratory data were collected for each patient. Clinical factors included age, history of epilepsy, cefepime use during admission, WHO severity of COVID-19 non-severe (asymptomatic, mild or moderate) disease, severe, and critical (18), concern for clinical seizure during admission or on arrival, structural injury on imaging, anoxic brain injury, laboratory values on admission including white blood cell count, glucose, sodium, hemoglobin, BUN, creatinine, lymphocyte count, D-dimer, erythrocyte sedimentation rate (ESR), ferritin, fibrinogen, aspartate transaminase (AST) and alanine transaminase (ALT), ammonia, dialysis, alcohol or substance use or withdrawal, sepsis, ARDS, clinical delirium, history of dementia, hypertension, COPD, obesity, CAD, treatment for COVID-19, renal failure, duration of illness prior to EEG, and pneumonia. All laboratory values analyzed were taken on the day of admission. Outcome measures included length of stay and discharge disposition. Associations with discharge disposition were analyzed by ordinal regression. Discharge options were home, acute rehabilitation, subacute rehabilitation, long-term acute care hospital and death. The electrographic characteristics of each patient's EEG were analyzed by experienced epileptologists. All EEGs were ordered either for clinical concern for altered mental status or seizure activity. Information extracted from EEG reports included background frequency, presence or absence of posterior dominant rhythm, focal slowing, any rhythmic, periodic or epileptiform discharges and seizure activity. Encephalopathy was defined as a background frequency of less than 8 Hz.

Statistical analysis was performed using SPSS. Associations between clinical variables and outcomes were initially determined by univariate linear or ordered logistic regression. Only clinical variables that were collected in all 50 subjects were included in the analysis. Multivariate analysis (stepwise regression) was then performed to determine if any of the variables that showed a significant correlation (*p* < 0.05) on univariate analysis were independently correlated with outcomes. Because there were 50 subjects, multivariate analysis was restricted to a maximum of five clinical variables with the lowest *p*-values on univariate analysis.

## Results

A total of 50 hospitalized non-ICU level patients with COVID-19 who underwent EEG studies were included from a single academic center. Their demographic and clinical characteristics are shown in Table 1. The average age of patients with EEG-defined encephalopathy was 70.7 years old (N = 23) and without encephalopathy was 61.5 years old (N = 27; *p* = 0.12). Female patients comprised 52% of patients. In total, 37 (74%) patients had non-severe WHO COVID-19 severity of illness, while 11 (22%) were severe, and 2 (4%) were critical. Of the patients without encephalopathy, 100% were mild in WHO classification in COVID severity. However, of the patients with encephalopathy, 25 (66%) were non-severe, 11 (29%) were severe, and 2 (5%) were critical. Of the clinical characteristics considered, only WHO severity scale distribution was statistically different between patients with and without encephalopathy after Bonferroni correction for multiple comparisons (Table 1).

**Table 1:** Patient characteristics.

**Clinical characteristics**	**Encephalopathy**	**No encephalopathy**	***p*-value**
	**(n = 23)**	**(n = 27)**	
Age, years, mean (SD)	70.7 (12.3)	61.5 (17.1)	0.12^†^
Female, n (%)	12 (52%)	16 (59%)	0.61
Structural injury on imaging, n (%)	12 (52%)	9 (33%)	0.18^‡^
Duration of illness prior to EEG, mean (SD)	8.4 (14.7)	5 (6.3)	0.63^†^
**WHO COVID severity scale, n (%)**			**0.002** ^**¥**^
Non-severe	14 (61%)	23 (85%)	
Severe	7 (30%)	4 (15%)	
Critical	2 (9%)	0 (0%)	
**Past Medical History, n (%)** ^‡^			
Dementia	9 (39%)	9 (33%)	0.67
Hypertension	18 (78%)	22 (81%)	0.78
Diabetes	11 (48%)	11 (41%)	0.61
Chronic obstructive pulmonary disorder	5 (22%)	4 (15%)	0.53
BMI > 30, kg/m^2^	8 (35%)	7 (26%)	0.5
Coronary artery disease	4 (17%)	7 (26%)	0.47
Epilepsy	6 (26%)	9 (33%)	0.58
**Clinical Course, n (%)** ^‡^			
Dialysis during hospitalization	3 (13%)	3 (11%)	0.83
Sepsis on admission	4 (17%)	2 (7%)	0.28
ARDS	3 (13%)	0 (0%)	0.053
Delirium present on admission	21 (91%)	17 (63%)	0.019
Cefepime	12 (52%)	5 (19%)	0.012
Pneumonia	15 (65%)	10 (37%)	0.047
Intubation	3 (13%)	0 (0%)	0.053
COVID-19 treatment	10 (43%)	3 (11%)	0.009
Renal failure	12 (52%)	7 (26%)	0.0567
Background frequency on EEG, mean (SD)	5.8 (1.1)	9.3 (1.3)	n/a
**Outcomes**			
Length of stay, mean (SD)	15.5 (9.9)	8.1 (7.3)	**0.001** ^‡^
**Disposition, n (%)**			**0.001** ^**¥**^
Death	3 (13%)	0 (0%)	
LTACH	1 (4%)	1 (4%)	
SAR	13 (57%)	7 (26%)	
AR	2 (9%)	3 (11%)	
Home	4 (17%)	16 (59%)	

ARDS, acute respiratory disease; BMI, body mass index; COVID-19, coronavirus disease 19; WHO, World Health Organization; SD, standard deviation. *P*-values in bold are significant after Bonferroni correction for 22 comparisons (corrected α = 0.0023).

† Two-sample Wilcoxon Rank-Sum test.

‡ Two-tailed Proportions test.

¥ Ordinal logistic regression.

**Figure 1:**
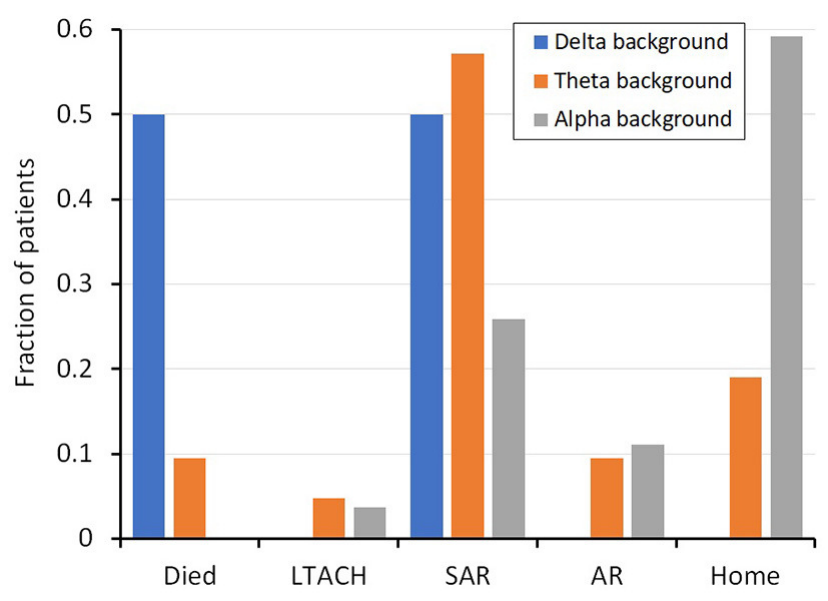
Fraction of patients with a given background frequency (i.e. alpha, theta, delta) and their discharge disposition. SD, standard deviation; SE, standard error measurement; LTACH, Long-term acute care hospital; SAR, subacute rehabilitation; AR, acute rehabilitation.

Outcome measures were significantly different between patients with and without encephalopathy. The length of stay was 7.4 days longer for those with encephalopathy (15.5 days) compared to those without (8.1 days; *p* = 0.001). Similarly, the discharge disposition was significantly different between subjects with or without encephalopathy (Table 1, Figure 1). Only 17% of patients with encephalopathy were discharged home, while 59% of those without encephalopathy were discharged directly home. Three patients, all of whom had encephalopathy on EEG, died in the hospital.

### EEG features in patients with encephalopathy

Varied EEG patterns were observed in this cohort. Background frequencies in the alpha range were noted in 27 (54%), in the theta range in 21 (42%), and 2 (4%) had a delta range background (1–4 Hz) (Table 1). None of the patients were on sedating medications.

Generalized periodic discharges with triphasic morphology (TW) were noted in 9 patients (18%). All these patients had a theta range background and were clinically reported to have altered mental status. Three patients (6%) had generalized periodic discharges without triphasic morphology. Generalized rhythmic delta activity was found in 5 patients (10%); all were superimposed on a background of theta range activity.

Focal findings included focal slowing, epileptiform discharges, periodic discharges, focal rhythmic activity or focal seizures (Table 2). Focal slowing was seen in 14 patients (28%), 5 of whom had structural pathology around the focus of slowing and 9 had no clinically significant imaging findings. Focal epileptiform discharges, including sharp waves, lateralized periodic discharges, and multifocal spikes, were noted in five (10%) patients. An analysis of findings from COVID-19 patients at this institution with epileptiform discharges and seizures with were previously reported in Santos de Lima et al. (18). Three of the five patients had a history of epilepsy and two had structural pathology that correlated with the location of the interictal findings. The other two patients had no clinically significant MRI findings.

**Table 2:** EEG features in Patients with COVID-19.

**EEG findings**	**N = 50**	**N**	**%**
Background	Alpha (8–12 Hz)	27	54
	Theta (4–8 Hz)	21	42
	Delta (1–4 Hz)	2	4
Generalized	Triphasic waves	9	18
findings	Generalized	3	6
	periodic discharges		
	Generalized rhythmic	5	10
	delta activity		
Focal findings	Focal slowing	14	28
	- Lesion	5	36
	- No lesion	9	64
	Focal interictal epileptiform activity	5	10
	- Lesion:	2	40
	LPDs	1	50
	Spikes	1	50
	- No lesion:	3	60
	LRDA	2	67
	Sharp wave	2	67
Seizures	Focal:	2	4
	- Lesion	1	50
	- No lesion	1	50
	Generalized	0	
	Status epilepticus	0	

LPDs Lateralized Periodic Discharges. LRDA Lateralized Rhythmic Delta Activity.

Two patients (4%) had focal electrographic seizures. One of the patients had focal structural pathology corresponding to the epileptogenic area and a known history of epilepsy. The second patient had no clinically significant structural pathology on imaging and had no history of epilepsy. No patient had a generalized seizure or was in status epilepticus, focal or generalized.

### Clinical associations with encephalopathy

We examined the association between a range of clinical variables and background frequency on EEG. Consistent with the use of low background frequencies as an electrographic marker of encephalopathy, the presence of clinical delirium was significantly associated with low background frequency (*p* = 0.004). Significant associations (*p* < 0.05) were also noted between the following binary (factor) variables and background frequency: cefepime use during admission (*p* = 0.007), pneumonia during admission (*p* = 0.028), and the presence of a structural lesion on imaging (*p* = 0.034). Univariate linear regression was used to assess the association between continuous variables and background frequency, and significant associations were found for age (inverse relationship; *p* = 0.017), WHO severity (*p* = 0.019), COVID treatment (*p* = 0.031) and BUN on the day of the admission (inverse relationship; *p* = 0.041). Twenty-two other clinical variables (see Methods for list of variables) were analyzed, but none were significantly associated with background frequency. Multivariate analysis that included delirium, cefepime use during admission, WHO severity, age, and pneumonia suggested that only clinical delirium (*p* = 0.000) and cefepime use (0.003) were independently associated with background frequency. Of the 17 patients who received cefepime, the average background frequency was 6.1 Hz, and of the 38 patients with delirium the average background frequency was 7.2 Hz.

### Patient outcomes

To determine if background frequency on EEG was an independently associated with patient outcomes, we considered other clinical parameters that might be related to length of stay in the hospital and disposition at discharge. Outcome analysis is outlined in Table 3.

**Table 3:** Clinical associations of encephalopathy, length of stay and outcome.

**Variable**	**T**	**95% CI**		***P***	**Variable**	**T**	**95% CI**		***P***
**Clinical associations of background frequency (N** = **50)**									
*Univariate analysis*					*Multivariate analysis*				
Delirium	−3.07	−3.238	−0.674	0.004	Delirium	−2.96	−2.842	−0.436	0.009
Cefepime use	−2.84	−2.824	−0.485	0.007	Cefepime use	−2.73	−2.422	−0.139	0.029
WHO severity	−2.7	−2.429	−0.357	0.009					
Age	−2.48	−0.081	−0.009	0.017					
Pneumonia	−2.26	−2.419	−0.141	0.028					
COVID treatment	−2.23	−2.738	−0.139	0.031					
Structural injury on imaging	−2.18	−2.412	−0.097	0.034					
BUN on the day of admission	−2.1	−0.057	−0.001	0.041					
**Clinical associations of length of stay (N** **=** **50)**									
*Univariate analysis*					*Multivariate analysis*				
Cefepime use	3.61	3.969	13.945	0.001	Cefepime use	3.14	2.804	12.845	0.003
Background frequency on EEG	−2.84	−2.881	−0.491	0.007					
Structural injury on imaging	2.49	1.199	11.362	0.016					
WHO severity	2.42	0.950	10.292	0.019					
Acute respiratory distress syndrome	2.16	0.816	22.234	0.035					
BUN on the day of admission	2.1	0.006	0.254	0.041					
Dialysis during admission	2.09	0.293	15.995	0.042					
Renal failure on admission	2.06	0.129	10.652	0.045					
**Clinical associations of outcome (N** **=** **50)**									
*Univariate analysis*	**Z**				*Multivariate analysis*	**Z**			
Acute respiratory distress syndrome	−2.67	−5.390	−0.823	0.008	Background frequency on EEG	3.49	0.272	0.968	0.000
Dialysis	−2.01	−3.482	−0.044	0.044					
Background frequency on EEG	3.67	0.284	0.937	0					

CI, confidence interval; Linear regression (T) for background frequency and length of stay; ordinal regression (Z) for outcome.

Clinical associations of length of stay were analyzed by linear regression. On univariate analysis, significant binary associations with length of stay (*p* < 0.05) included receiving at least one dose of cefepime during admission (*p* = 0.001), structural injury on imaging (*p* = 0.016), acute respiratory distress syndrome (*p* = 0.035), dialysis (*p* = 0.042), and renal failure (*p* = 0.045). The significant continuous associations with length of stay (*p* < 0.05) were background frequency (*p* = 0.007), WHO severity of COVID-19 (*p* = 0.019), and BUN on the day of admission (*p* = 0.025). Multivariate analysis of the significant univariate factors that included cefepime use on admission, background frequency, ARDS, WHO severity and structural injury on imaging showed that only cefepime use during admission (*p* = 0.003) was independently associated with length of stay. Of the 17 patients who received cefepime, two had clinical pneumonia with septic shock and died during the hospitalization.

Significant associations with outcome on univariate analysis were ARDS (*p* = 0.008), dialysis (*p* = 0.044), and background frequency (*p* = 0.000). Multivariate analysis that included these three variables showed that only background frequency on EEG was independently associated with discharge disposition (*p* = 0.000).

## Discussion

Encephalopathy in COVID-19 has been the topic of much interest since the start of the pandemic. We retrospectively reviewed 50 non-ICU level hospitalized patients with a positive nasal swab for SARS-CoV-2 virus and an EEG, characterizing the clinical associations with encephalopathy, length of stay and discharge disposition.

The observed correlation between low background frequency and clinical delirium is in line with previous studies that suggest background frequency is a sensitive marker of encephalopathy (19, 20). While WHO severity scores were different in patients with or without electrographically defined encephalopathy, multi-variate analysis suggests that clinical delirium and cefepime use were the only variables independently associated with background frequency.

Only cefepime use was significantly associated with length of stay on multivariate analysis. Cefepime and piperacillin-tazobactam are intravenous, broad-spectrum, antibiotics often used for empiric coverage of possible pseudomonal infection in clinically deteriorating patients. Use of either cefepime or piperacillin-tazobactam as primary anti-pseudomonas coverage varies by institution based on internal guidelines; at the University of Chicago cefepime is the default while piperacillin-tazobactam is only rarely used. Each option has potential side effects, and cefepime has been associated with encephalopathy in a small fraction of patients, especially those critically ill with renal impairment (21, 22). The duration of empiric therapy is typically 7–10 days, but antibiotics are often deescalated after 3 days if suspicion of infection resolves. The prolonged stay for patients who received cefepime likely reflects the need for 3–10 days of in-house IV therapy, and cefepime use is likely a surrogate marker for bacterial superinfection, a well-described complication of COVID-19 (23, 24).

Finally, background frequency on EEG was the only variable with a significant association with discharge disposition in a multivariate analysis. Since background frequency is a quantitative measure of brain function, it is not surprising that it is correlated with disposition. However, it was unexpected that it was better associated with disposition than COVID severity of illness (WHO categorization) or pulmonary symptoms. This finding implies that of the varied effects of COVID, the short-term outcome is most closely related to its effect on cognition. Previous studies have shown that even mild COVID infection can affect cognitive function 6 months after infection, although EEG background frequency is not sensitive to the relatively mild cognitive dysfunction at this time point (25). For those who recover from mild COVID infections, cognitive function tends to recover many months later, returning to baseline after 18 months (26). In the short term, therefore, EEG changes were associated with hospital disposition, cognitive changes remain in the first 6 months although EEG abnormalities tend to resolve, and cognitive changes return to baseline within a year and a half.

An important limitation on the interpretation of this study is due to an inherent selection bias: patients were only included if they had a clinical indication for an EEG. It is likely that the population studied has different characteristics than the general COVID population, so it is not known whether the results would generalize if all patients underwent EEGs. In addition, the patient population only included those infected with SARS-CoV-2 variants before omicron; omicron and subsequent variants appear to have a slightly different clinical course (27). Additionally, the sample size was small with only 50 subjects, limiting the ability to include more than five variables in multi-variate analysis. Finally, patient outcome measures did not extend past discharge, so it is not clear how acute EEG changes relate to the post-COVID cognitive syndrome known as long COVID (28). Prospective studies with a larger sample size could address these limitations, especially as they relate to long COVID.

## Data availability statement

The raw data supporting the conclusions of this article will be made available by the authors, without undue reservation.

## Ethics statement

Ethical review and approval was not required for the study on human participants in accordance with the local legislation and institutional requirements. Written informed consent for participation was not required for this study in accordance with the national legislation and the institutional requirements.

## Author contributions

KS, WL, AEi, SK, and NI contributed to writing. KS, WL, AEi, and AEs collected clinical data. SA and JT reviewed EEG patterns. WL and NI performed statistical analysis. All authors contributed to the article and approved the submitted version.

## Funding

NI is supported by NIH grant 1R01NS116262.

## Conflict of interest

The authors declare that the research was conducted in the absence of any commercial or financial relationships that could be construed as a potential conflict of interest.

## Publisher's note

All claims expressed in this article are solely those of the authors and do not necessarily represent those of their affiliated organizations, or those of the publisher, the editors and the reviewers. Any product that may be evaluated in this article, or claim that may be made by its manufacturer, is not guaranteed or endorsed by the publisher.
